# Heterogeneity and molecular landscape of melanoma: implications for targeted therapy

**DOI:** 10.1186/s43556-024-00182-2

**Published:** 2024-05-10

**Authors:** Yasaman Zohrab Beigi, Hossein Lanjanian, Reyhane Fayazi, Mahdieh Salimi, Behnaz Haji Molla Hoseyni, Mohammad Hafez Noroozizadeh, Ali Masoudi-Nejad

**Affiliations:** 1 Laboratory of System Biology and Bioinformatics (LBB), Institute of Biochemistry and Biophysics, University of Tehran, Tehran, Iran; 2 Software Engineering Department, Engineering Faculty, Istanbul Topkapi University, Istanbul, Turkey; 3 Department of Medical Genetics, Institute of Medical Biotechnology, National Institute of Genetic Engineering and Biotechnology (NIGEB), Tehran, Iran; 4 Negah Eye Hospital, Tehran, Iran

**Keywords:** Uveal melanoma (UM), Heterogeneity, Targeted therapy, Liquid biopsy, Circulating tumor cells (CTCs), Single-cell analysis

## Abstract

Uveal cancer (UM) offers a complex molecular landscape characterized by substantial heterogeneity, both on the genetic and epigenetic levels. This heterogeneity plays a critical position in shaping the behavior and response to therapy for this uncommon ocular malignancy. Targeted treatments with gene-specific therapeutic molecules may prove useful in overcoming radiation resistance, however, the diverse molecular makeups of UM call for a patient-specific approach in therapy procedures. We need to understand the intricate molecular landscape of UM to develop targeted treatments customized to each patient's specific genetic mutations. One of the promising approaches is using liquid biopsies, such as circulating tumor cells (CTCs) and circulating tumor DNA (ctDNA), for detecting and monitoring the disease at the early stages. These non-invasive methods can help us identify the most effective treatment strategies for each patient. Single-cellular is a brand-new analysis platform that gives treasured insights into diagnosis, prognosis, and remedy. The incorporation of this data with known clinical and genomics information will give a better understanding of the complicated molecular mechanisms that UM diseases exploit. In this review, we focused on the heterogeneity and molecular panorama of UM, and to achieve this goal, the authors conducted an exhaustive literature evaluation spanning 1998 to 2023, using keywords like "uveal melanoma, “heterogeneity”. “Targeted therapies”," "CTCs," and "single-cellular analysis".

## Introduction

Uveal melanoma (UM), constituting the most common primary intraocular malignancy in adults, is a rare tumor accounting for less than 5% of all melanoma cases. It predominantly affects the choroid (90%), followed by the ciliary body (6%) and iris (4%). In the United States, the age-adjusted risk of uveal melanoma is estimated at 5.1 per million individuals, with Caucasians exhibiting a higher incidence. Typically diagnosed around the age of 62, uveal melanoma's prevalence is expected to rise alongside the aging population. Given the anticipated increase in ocular conditions, timely identification and effective management of such ailments are crucial for preserving visual health and improving overall quality of life. This malignancy originates in the uveal tract of the eye, encompassing the iris, ciliary body, and choroid, where pigment-producing melanocytes reside. Usually, uveal melanoma begins within these melanocytes, with the choroid serving as its primary site of development [1–3].

**Fig. 1:**
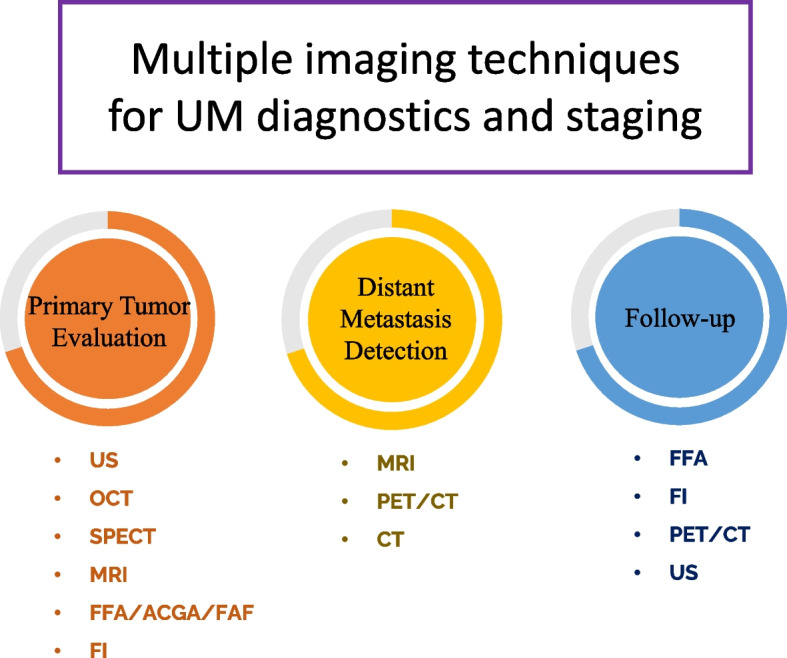
Preferred Imaging Modalities for Uveal Melanoma Diagnosis and Follow-up. These imaging modalities play a crucial role in the comprehensive management of uveal melanoma patients, enabling clinicians to accurately assess tumor characteristics, such as size, location, and growth patterns. Additionally, these imaging techniques facilitate the early detection of metastasis and guide treatment decisions, ensuring timely interventions for optimal patient outcomes. (Abbreviations: SPECT: single-photon emission computed tomography; FFA: fundus fluorescein angiography; OCT: optical coherence tomography; MRI: magnetic resonance imaging; US: ultrasonography; ICGA: indocyanine green angiography; FAF: fundus autofluorescence; PET: positron emission tomography; CT: computed tomography.)

Advancements in local treatments for primary UM have improved outcomes, with a focus on conservative approaches to preserve ocular function. However, the prognosis for patients with metastatic disease remains poor, with persistently low 5-year survival rates, even with the improvements in localized disease management [4–6]. Metastatic screening, primarily through liver imaging using ultrasound, MRI, and liver function tests, is recommended following a UM diagnosis. While less than 4% of patients have detectable metastatic disease at diagnosis, approximately 50% develop metastases within a few years. The liver is the most common site of metastasis, followed by the lungs and bones. Metastatic UM is associated with high fatality rates, with the majority of patients succumbing to the disease within one to two years after the diagnosis of metastases [2, 3, 7, 8]. Figure 1 outlines the preferred imaging techniques utilized at various stages of uveal melanoma diagnosis and follow-up, providing a comprehensive overview of clinical practice.

Prognostic indicators for UM include demographic, clinical, and histological characteristics, such as age, gender, tumor size, location, configuration, staging, and specific histopathological features [9–11]. In recent years, the role of genetics in prognostication has gained prominence. Fine-needle aspiration biopsy (FNAB) is commonly used for molecular analysis during plaque brachytherapy, but other sampling techniques, such as incisional biopsy, may be employed based on tumor characteristics. Clinical and ultrasonographic features may be atypical due to racial pigmentation necessitating diagnostic FNAB. This integration of genetic analysis with different biopsy techniques allows for a comprehensive evaluation of the tumor, enabling more accurate prognostication and personalized treatment strategies [12–14]. However, all biopsy techniques carry the risk of complications and potential vision-threatening effects [14, 15]. Alternative methods, like whole genome amplification and sequencing, offer promise for accurate prognostic testing, overcoming the limitations of FNAB [1, 12, 16, 17]. It is essential to consider the psychological implications for patients undergoing prognostic testing, as they desire information despite limited treatment options for metastatic disease. Managing UM involves tailoring treatments to tumor characteristics, with local treatments advancing. However, metastatic UM remains challenging with a grim prognosis. Prognostic indicators and genetic testing are vital for risk assessment and treatment choices. Tumor sampling methods, like FNAB and alternatives, have risks and limitations. Recognizing the psychological impact of prognostic testing is essential for holistic patient care [18–20].

This review explores recent advancements in the Heterogeneity and Molecular Landscape of Uveal Melanoma, with a focus on its implications for targeted therapy. It investigates the immunological heterogeneity and therapeutic approaches in uveal melanoma, alongside its detection using liquid biopsy techniques, particularly single-cell analysis and circulating tumor cells (CTCs). Emphasizing the significant potential of CTCs as a non-invasive tool, the review elucidates their role in prognosis, disease monitoring, and therapeutic decision-making. By offering a comprehensive analysis, this review illuminates the profound impact of single-cell analysis on our understanding of uveal melanoma and underscores its potential clinical significance in addressing this challenging disease.

## Heterogeneity in melanoma

In UM, intra-tumoral heterogeneity is a prevailing phenomenon tightly linked to genomic instability, resulting in the emergence of distinct subclone populations within the tumor cell population, particularly evident in medium- and large-sized tumors. Morphological heterogeneity is observed in UM specimens, with mixed cellularity presenting both epithelioid and spindle cell patterns in varying proportions. The most common forms of uveal melanoma are mixed epithelioid-spindle cell tumors, which represent 48% of all cases, followed by spindle-B cell tumors (32%). The other forms of the tumor, such as necrotic, spindle-A, fascicular, and epithelioid, are less common. Clinical and histologic features like microvascular loops, vascular mimicry, epithelioid cell type, and the presence of tumor-infiltrating lymphocytes and macrophages are identified as risk factors in UM [21, 22]. The evaluation of chromosomal and genetic modifications has become crucial in predicting prognosis for UM, with prevalent driver mutations identified in genes GNAQ or GNA11, and a smaller subset in PLCB4 or CYSLTR2. Despite the initial stages of direct targeting of oncoproteins resulting from these mutations, targeted therapy utilizing BRAFV600E has demonstrated efficacy in treating cutaneous melanoma. Mutations in UM driver genes impact common downstream signaling pathways, including PKC/MAPK, PI3K/AKT, and YAP/TAZ, which are regarded as actionable targets. Loss of BAP1, a characteristic of UM metastasis, affects chromatin structure through histone H2A deubiquitylation, potentially reversible via histone deacetylase inhibitors. Preclinical investigations have identified potential advantages of targeting various pathways, encompassing Bcl-2, histone deacetylase, ubiquitin–proteasome, phosphatidylinositol-3-kinase-AKT, adhesion molecules, mitogen-activated protein kinase, receptor tyrosine kinases, matrix metalloproteinase, and angiogenic factors. Clinical trials are underway to explore these approaches in patients with metastatic uveal melanoma, as well as in the adjuvant setting following primary therapy [23–25]. Targeted therapy, which involves medications disrupting specific molecular pathways vital in tumor development or progression, presents a precise approach distinct from conventional cytotoxic chemotherapy and immune-based treatments. Hormonal therapies for breast and prostate cancer exemplify this approach. Recent progress in comprehending the molecular mechanisms underlying cancers has ushered in a new era of therapeutic agents. These include drugs that modulate pathways governing cell cycle regulation, apoptosis, proliferation, invasion, metastasis, and angiogenesis the critical step controlling tumor growth and spread. Consequently, several targeted therapeutics have obtained approval for treating cancers that were previously resistant to treatment [14, 26, 27].

Based on the diverse findings outlined in this study, it is possible to present the following comprehensive classification of therapeutic approaches for the treatment of melanoma. These findings shed light on potential methods that merit careful consideration in addressing this particular medical condition [28]. Local Treatments containing, Uveal melanoma Radiotherapy [29], Transpupillary Thermotherapy (TTT), Surgical Interventions including Enucleation [30–32] and Resection of Tumor [33–36], Emerging Approaches by Photodynamic Therapy (PDT) [37–39], and Local Drug Delivery [40, 41], Systemic Therapies containing Targeted Therapies [42–44] and Immunotherapies [45, 46]. Overall insight, is shown in Table 1.

**Table 1:** Different methods are used for treating uveal melanoma. These methods are known as therapeutic modalities [47]

Therapy	Primary UM	Advanced UM
Enucleation (Removal of the eye)	No	Yes
Exenteration	No	Yes
Local resection	No	Yes
Plaque brachytherapy	Yes	Yes
Proton beam therapy	Yes	Yes
Transpupillary thermal therapy	Yes	No
Photodynamic therapy (PDT)	Yes	Yes
Stereotactic radiosurgery	No	Yes
Gamma knife radiosurgery	Yes	Yes
Cyberknife radiosurgery	Yes	Yes
Linear accelerator	Yes	Yes
Charged particle radiation therapy	Yes	Yes
Systemic chemotherapy	No	Yes
Anti-CTLA-4 antibodies	No	Yes
Anti-PD-1 antibodies	No	Yes
Bispecific molecule	No	Yes
Adoptive T-cell therapy	No	Yes
Molecular targeted therapy	No	Yes
Liver directed therapies	No	Yes

### Definition and significance of heterogeneity

There are different sources of intra-tumoral heterogeneity (ITH), such as clonal evolution, tumor cell plasticity (stem cell formation), and heterogeneity in the tumor microenvironment [48–50]. From a molecular point of view, differences in the genetic, epigenome, transcriptome, and proteome of tumor cells will result in the tumor's cell-to-cell variation in spatial and temporal modes [51, 52]. Genomic instability, interactions with the tumor microenvironment, and evolutionary selection pressures contribute to heterogeneity. Different types of therapeutic strategies, such as chemotherapy and targeted therapy, can influence intra-tumoral heterogeneity. This, in turn, affects resistance to therapy by directly affecting the molecular spectrum and selection pressure. This resistance can be a consequence of preexisting heterogeneity, or ongoing emergence of ITH [53]. So, ITH serves as an obstacle in cancer therapy. For example, Genotype-guided therapy, a subtype of targeted therapy, translates ITH into clinical practice and contributes to tumor therapy resistance [52]. Moreover, immunotherapy can be influenced by ITH, and the response to immune checkpoint blockers can be predicted by ITH [53, 54].

### Molecular and phenotypic heterogeneity in melanoma

Molecular (genetic and epigenetic) and cellular heterogeneities have been known for Uveal Melanoma. Genetic heterogeneity is now evident in different driver genes (BAP-1, SF3B1, EIF1AX, GNQ, and GNA11) and subsequent mutations, as well as chromosomal copy number alterations (in chromosomes 1, 3, 6, 8, and 16) [55, 56]. These genomic alterations are present in both Class 1 GEP and Class 2 GEP, albeit at different rates [57]. In addition, immunohistochemical analysis confirmed the presence of spatial epigenetic intra-tumoral heterogeneity (H3Ac, H4Ac, 5-MeC, 5-hMeC, UBC, and H2Aub) in UM [58].

Field et al. proposed that UM undergoes punctuated tumor evolution, with genomic aberrations occurring early in time. They deduced that the potential for metastasis is an inherent property that precedes the clinical detection of the primary tumor [59]. N. Smith et al. also have demonstrated this pattern for epigenetic changes like methylation. Through genome-wide methylation analysis technique, They demonstrated no novel shared methylation pattern among different metastasis, implying that metastatic events also occurred early in UM evolution time [60].

Indeed, cellular heterogeneity is attributed to the presence of non-malignant cells, mostly immune cells, in the tumor microenvironment [55]. A single-cell transcriptomic analysis of immune cells revealed heterogeneity in myeloid and lymphoid cells, their phenotypes, and the clonal diversity of T cells [61]. Li et al. have demonstrated the heterogeneity among macrophages in the UM tumor microenvironment by identifying four different subtypes of macrophages [62]. With the help of bulk or single-cell bioinformatic-based analysis of T T-cell receptors (TCR) repertoire heterogeneity, it is evident that there is spatial heterogeneity in T-cell density and clonality. This heterogeneity is proportional to tumor ubiquitous and regional neoantigen loads. While accessible through blood sampling, the analysis of ubiquitous TCRs can enhance personalized immunotherapies [62–64]. TCR analysis for UM is limited, except for the work conducted by Huuhtanen et al. as part of a study on cutaneous melanoma [65].

These heterogeneities of UM have been identified using experimental tools, next-generation sequencing data, bioinformatic tools, and digital PCR. They have been confirmed and further developed through single-cell-based analysis.

### Tumor microenvironment and its influence on heterogeneity

The interconnection between the tumor microenvironment and intra-tumoral heterogeneity is complex. This complexity arises from both spatial and phenotypic heterogeneity in immune cells (referred to as immune-ITH) within the tumor microenvironment and its relation to the heterogeneity of tumor cells. According to Nguyen et al., the impact of immune-ITH on tumor cell ITH (including genomic and transcriptomic heterogeneity) is evident. Through bulk genomic and transcriptomic analysis, they demonstrated the influence of immune-ITH on tumor evolution and immunoediting [66].

Indeed, the reciprocal negative relationship between the ITH of tumor cells and immunosurveillance adds to this complexity. The stronger the immunosurveillance, the less heterogeneous a tumor is, as immunological subclones are eliminated. Conversely, the more heterogeneous a tumor is, the more it evades immune response. Dijkstra et al. suggested that this phenomenon may be related to various factors, such as immunodomination, competition, antigen dosage, and the induction of harmful responses as collateral damage [67].

## Molecular landscape of melanoma

### Key genetic mutations and alterations in melanoma

**Fig. 2:**
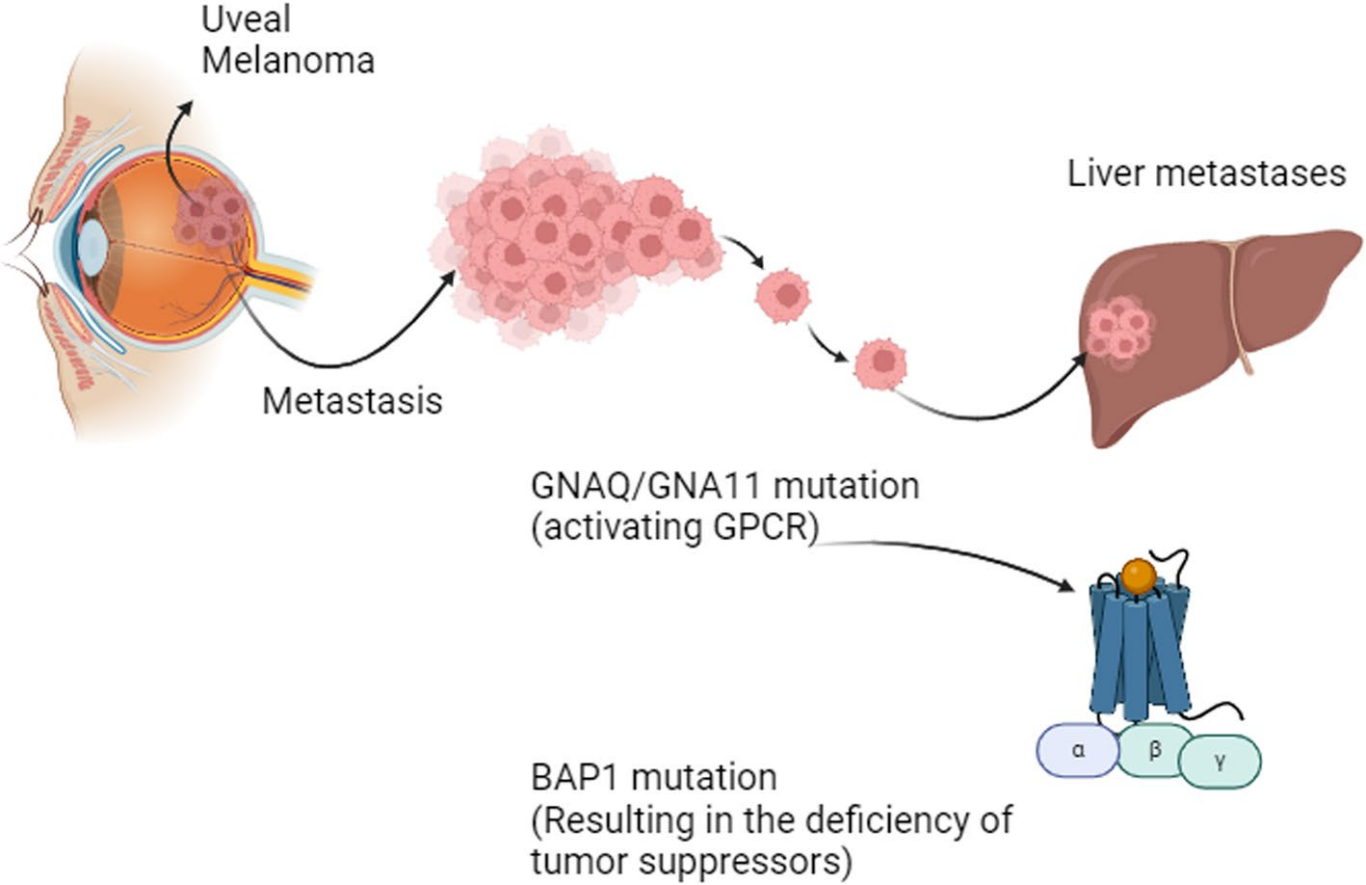
Overview of Metastatic Uveal Melanoma to the Liver with Main Mutations (GNAQ/GNA11 and BAP1 mutations). This figure provides a comprehensive overview of metastatic uveal melanoma specifically focusing on its dissemination to the liver, which is a common site of metastasis for this type of cancer. It highlights the key genetic mutations associated with metastatic uveal melanoma, notably mutations in the GNAQ/GNA11 genes and the BAP1 gene. The dysregulation of G protein-coupled receptor (GPCR), stemming from mutations in GNAQ/GNA11 genes, initiates oncogenic signaling pathways, including MPAK, PI3K/AKT, or YAP/TAZ, thereby promoting tumor progression. These mutations are known to contribute to the pathogenesis of uveal melanoma and are associated with a deficiency in tumor suppressor activity, promoting tumor growth and metastasis. (Created with BioRender.com)

Uveal melanoma, a malignancy of the eye, is affected by several risk factors, including fair skin, blonde hair, light eye color, the presence of choroidal nevus, and the germline mutation in the breast cancer 1-associated protein 1 (BAP1) [20, 68–70]. The role of ultraviolet exposure in uveal melanoma risk remains a subject of debate and requires further investigation. However, advancements in molecular biomarker identification and characterization have greatly contributed to the detection and understanding of this disease. Extensive research has uncovered recurrent genetic alterations in uveal melanoma, with mutations in SF3B1, BAP1, CYSLTR2, GNAQ, GNA11, and PLCB4 being particularly prevalent. These genetic abnormalities hold significant clinical relevance as they play a crucial role in uveal melanoma pathogenesis. Conversely, the absence of mutations in EIF1AX, associated with a favorable prognosis in uveal melanoma, has been noted. The comprehension and utilization of these molecular biomarkers offer promising avenues for enhanced diagnostics and personalized therapeutic strategies in the management of uveal melanoma patients [71–74]. Figure 2 presents an overview of metastatic uveal melanoma in the liver, highlighting key mutations associated with this progression.

To date, there is a lack of effective treatment options available for patients with metastatic UM. The median overall survival (OS) time for these patients is approximately 10 to 13 months, and the chances of a complete cure are exceedingly low [75]. Consequently, the primary clinical objective currently revolves around improving the survival rates for individuals with metastatic UM. In pursuit of this goal, several studies have dedicated efforts to identify prognostic biomarkers that can provide valuable insights. For instance, Wang et al. conducted research that led to the discovery of MMP1 and MMP9 as potential biomarkers with the ability to predict UM OS and disease-free survival [76–78]. These findings offer hope for the development of novel strategies and interventions to enhance the prognosis and outcomes for patients facing this challenging condition.

Distant metastasis is infrequent at the time of initial ocular presentation, occurring in less than 5% of cases. Treatment of the primary tumor typically involves localized therapies aimed at preserving the eye, such as laser or radiation, with enucleation as an alternative option [27, 79]. Prognostic factors for predicting metastatic risk include cytogenetics, RNA-based gene expression profiling, mutational analysis, and elevated expression of the ABCB5 protein, which is associated with a poor prognosis and an increased risk of metastasis. Following primary tumor management, routine surveillance scans, including abdominal imaging (CT or MRI), are recommended every 3 to 6 months [80–83].

Prognostic markers play a pivotal role in the prediction of clinical outcomes and disease progression in uveal melanoma, a complex ocular malignancy. Extensive research has identified various molecular and genetic factors that hold potential as prognostic markers in this context [84, 85]. These factors encompass chromosomal abnormalities, gene mutations (such as GNAQ and GNA11), gene expression profiles, tumor size, tumor location, and histopathological features. Notably, specific biomarkers like monosomy 3 and gains in chromosome 8q have demonstrated associations with an increased risk of metastasis and poorer prognosis [17, 86–90]. The identification and validation of these prognostic markers offer valuable insights into risk stratification, treatment selection, and the ongoing monitoring of patients with uveal melanoma. Consequently, this knowledge facilitates the development of personalized management strategies aimed at optimizing patient outcomes. In a conducted study, a meticulous analysis of 80 uveal melanomas was executed, encompassing the strategic classification of poor-prognosis monosomy 3 UM into discrete subsets predicated upon diverse genomic aberrations, transcriptional attributes, and clinical prognoses. This investigative endeavor delved profoundly into DNA methylation profiles and somatic copy number alterations, culminating in the efficacious stratification of more favorable-prognosis disomy 3 UM into risk groups of low or intermediate nature. Noteworthy is the revelation that both disomy 3 (D3) and monosomy 3 (M3) UM evinced intricate molecular substructures, each manifesting an array of clinical outcomes. Particularly salient is the identification of a distinctive global DNA methylation pattern exclusively evident in poor-prognosis M3-UM. Furthermore, within the ambit of poor-prognosis M3-UM, distinct subsets surfaced, characterized by unique genomic, signaling, and immune profiles. The study also brought to the fore discernible variances in genomic and DNA methylation profiles between EIF1AX and SRSF2/SF3B1 mutant D3-UM, signifying the nuanced intricacies within this genomic landscape [91–93]. Table 2 showcases the subtypes of uveal melanoma along with their associated symptoms or patient complaints, providing a comprehensive overview of these distinct classifications.

**Table 2:** Subtypes of Uveal Melanoma and Corresponding Symptoms or Patient Complaints

Subtype	Related Symptoms or Patient Complaints
Spindle Cell	- Blurred or distorted vision
	- Floaters in the vision
Epithelioid Cell	- Decreased visual acuity
	- Eye pain
Mixed Cell	- Vision loss
	- Distorted or wavy vision
	- Photopsia (flashes of light)

### Signaling pathways involved in melanoma development and progression

Most cases of UM are caused by mutations in the GNAQ or GNA11 genes, while a smaller percentage of tumors result from mutations in the PLCB4 or CYSLTR2 genes. Currently, there are no direct inhibitors available that target the oncoproteins produced by these mutations. This is in contrast to cutaneous melanoma, where targeted therapy has shown significant success. However, UM driver mutations converge on common downstream signaling pathways such as PKC/MAPK, PI3K/AKT, and YAP/TAZ. Therefore, these pathways are considered as actionable targets for treatment [72, 94–99].

The absence of BAP1 is a significant contributor to metastasis in uveal melanoma. This deficiency disrupts chromatin organization by influencing histone H2A deubiquitination, a process that may be reversed through the use of histone deacetylase inhibitors. While preclinical investigations targeting signaling molecules like MAPK and PKC showed promising results, their effectiveness was not confirmed in initial clinical trials. A thorough review of all clinical trials involving novel targeted and immune therapies for uveal melanoma unveiled disappointing outcomes [100–102].

Primary UM is characterized by recurring chromosome abnormalities in chromosomes 1, 3, 6, 8, 9, and 16. These genetic changes have a significant impact on the prognosis of patients and are crucial in classifying them into different risk groups. The most common chromosomal aberrations in primary UM are the loss of 1p (28–34%), a gain of 1q (24%), loss of 3 (50–61%), gain of 6p (28–54%), gain of 6q (28–54%), loss of 6q (35–37%), loss of 8p (17–28%), gain of 8q (36–63%), loss of 9p (24%), and loss of 16q (16%) [103–106].

It has been found that the occurrence of monosomy 3 and the gain of chromosome 8q in tumors is linked to an increased risk of metastasis. When tumor cells have a higher proportion of monosomy 3 and gain of 8q, it directly correlates with a poorer prognosis [103, 106–108]. Research indicates that the loss of chromosome 8p is associated with a faster onset of metastasis, indicating increased metastatic efficiency. On the other hand, the gain of chromosome 6p is linked to a favorable prognosis, suggesting a protective effect. Additionally, the benefit associated with 6p gain may be due to its mutual exclusivity with monosomy 3 [109, 110].

It seems that Monosomy 3 is an early occurrence in the development of uveal UM. On the other hand, the loss of 1p and 8p, as well as the gain of 8q, are considered secondary events in UM progression, especially about larger tumor sizes [108, 111, 112].

UM is classified based on both cytogenetic characteristics and gene expression profiling. Gene expression profiling involves analyzing RNA levels from a 15-gene panel. The DecisionDx-UM Gene Expression Profile, developed by Castle Biosciences, is a commercial test that uses 12 discriminating genes (ECM1, CDH1, EIF1B, FXR1, HTR2B, LTA4H, ID2, LMCD1, ECM1, MTUS1, RAB31, SATB1, and ROBO1) and 3 control genes (RBM23, MRPS21, and SAP130) to categorize tumors into Class 1A and 1B (low risk) and Class 2 (high risk of metastasis). This test is conducted on a microfluidics platform [56, 111, 113, 114].

Class 1A tumors are considered to be at "very low risk" of metastasis within 5 years, with only a 2% probability. Class 1B tumors are categorized as "low risk" with a 21% chance of metastasis within 5 years. Class 2 tumors are considered "high-risk" with a 72% risk of metastasis within 5 years. A Collaborative Ocular Oncology Group study validated the prognosis and indicated that the assay might perform better than the chromosome 3 status in clinical prognostic testing [115, 116]. A recent study was conducted on 89 patients at four different centers. The study revealed that Class 1 tumors had a metastasis rate of 10%, while Class 2 tumors had a much higher rate of 58%. It was also found that the metastasis-free survival rate for Class 1 tumors was 90%, whereas it was only 41% for Class 2 tumors over a period of 5 years [115, 117].

The gene expression profiles (GEP) of Class 1 tumors exhibit similarities to those of normal uveal melanocytes and low-grade uveal melanocytic tumors, whereas the GEP of Class 2 tumors bears resemblance to primitive neural or ectodermal cells [118, 119]. Class 1 tumors frequently harbor mutations in EIF1AX and SF3B1, whereas Class 2 tumors are often linked to mutations in the BAP1 tumor suppressor gene [7, 115, 120]. There is a notable association between Class 1 tumors and disomy 3, as well as between Class 2 tumors and monosomy 3. In 20.8% of cases, there was a discrepancy between the outcomes derived from the GEP and chromosome 3 tests [115, 116, 121].

In recent studies, researchers have investigated the expression of messenger RNA from a cancer-testis antigen known as PRAME (Preferentially Expressed Antigen in Melanoma), alongside GEP. PRAME expression is recognized as an independent biomarker, adding an extra dimension of prognostic precision to the Class 1/Class 2 GEP system [115, 120]. Table 3 illustrates the genetic mutation-based classification of uveal melanoma into four distinct classes: A, B, C, and D. This classification system is instrumental in understanding the molecular heterogeneity of uveal melanoma, allowing for precise categorization based on genetic alterations.

**Table 3:** The Cancer Genome Atlas (TCGA) Classification System: Genetic Mutation-Based Classification of Uveal Melanoma into Classes A, B, C, and D. (TCGA classification system emerged from a collaborative effort spearheaded by the National Cancer Institute Center for Cancer Genomics and the National Human Genome Research Institute in 2005. The TCGA project was established with the primary objective of comprehensively characterizing the molecular alterations inherent in cancer cells through the analysis of vast amounts of data acquired from numerous human samples. This ambitious initiative aimed to enhance our understanding of the intricate molecular landscape of cancer and provide valuable insights into the underlying mechanisms driving tumorigenesis and disease progression) [8, 9, 14, 122]

**Characteristic Genetic Aberrations per TCGA Class**	**TCGA A/GEP class 1 A (~ 45%)** Partial or total gain of 6p	**TCGA B/GEP class 1 B (~ 20%)** Gain of 6p, partial 8q gain	**TCGA C/GEP class 2 (~ 25%)** Gain of 8q	**TCGA D/GEP class 2 (~ 10%)** Amplification of 8q
Chromosome 3	Disomy 3/GEP class 1 tumors	Disomy 3/GEP class 1 tumors	Monosomy 3/GEP class 2 tumors	Monosomy 3/GEP class 2 tumors
Chromosome 8	Normal 8q	8q gain	8q gain	8q gain (Multiple)
Significantly mutated genes	EIF1AX	SF3B1 or SRSF2	BAP1	BAP1
Prognosis	Favorable	Late metastasis	Unfavorable	Unfavorable

*Abbreviations*: *BAP1* BRCA1 Associated Protein 1, *SF3B1* Splicing Factor 3b Subunit 1, *EIF1AX* Eukaryotic Translation Initiation Factor 1A X-Linked

### Role of immune system in melanoma

Primary and liver metastatic UM reside in environments that are immune-privileged and immunologically tolerant organs, respectively. Immune-privileged sites, less likely to induce an immune response to new antigens, are protected by physical and functional barriers to circulating effector immune cells to inhibit destructive inflammation [123]. While there is a statement that epigenetic modification of tumor cells in privileged organs may result in emerging tumor cells that can easily escape the immune attack, others believe that less immune edition will occur in privileged organs [124, 125]. The liver, an immunological organ highly exposed to circulating antigens, masters at suppressing the immune response to induce tolerance in homeostasis [126, 127]. This fact along with other characteristics, makes the liver a favorable site for primary and metastatic tumor cells to grow. Additionally, the less mutational burden of UM makes it less likely to be attacked by the immune system and also immunotherapy.

When unraveling the immune system landscape in UM, it is important to consider immune cells in both the tumor microenvironment (TME) and circulation. There are some attempts to Characterize the tumor immune microenvironment and evaluate potential responses to immunotherapy like Immune checkpoint blocker (ICB) in UM. Goesmann et al. have tried to find a more detailed understating of primary UV TME with the help of Immunohistochemistry in examining immune cell localization. They found that immune cells most likely reside in the outer section of tumors. Interestingly CD68 immunosuppressive macrophages and CD3 immunosuppressive T cells showed similar localization to tumor cells but with different frequencies in tumors of different parts of the eye. Just like immune cell localization, it has been done for immune checkpoint molecules like LAG-3, LSECtin, and Galectin-3 [128]. Mariani et al. demonstrated that tumor-infiltrating macrophages were more prevalent in liver metastases compared to primary uveal melanoma. However, tumor-infiltrating lymphocytes were scarcely seen in both primary and liver metastatic UM. Their univariate survival-related analysis indicated that CD68 + , CD163 + macrophages, and CD20 + B cells had a positive influence on overall survival and metastasis-specific overall survival [128, 129]. There is more trafficking of lymphocytes and macrophages in tumors with monosomy of chromosome 3p [113, 130]. Lymphocytes in metastatic UM are functionally dormant, as demonstrated by the concordance presence of CD8 + T cells, CD25 and/or FoxP3-positive T cells, and PD1 expression in tumor-infiltrating lymphocytes through immunohistochemical analysis [131].

**Fig. 3:**
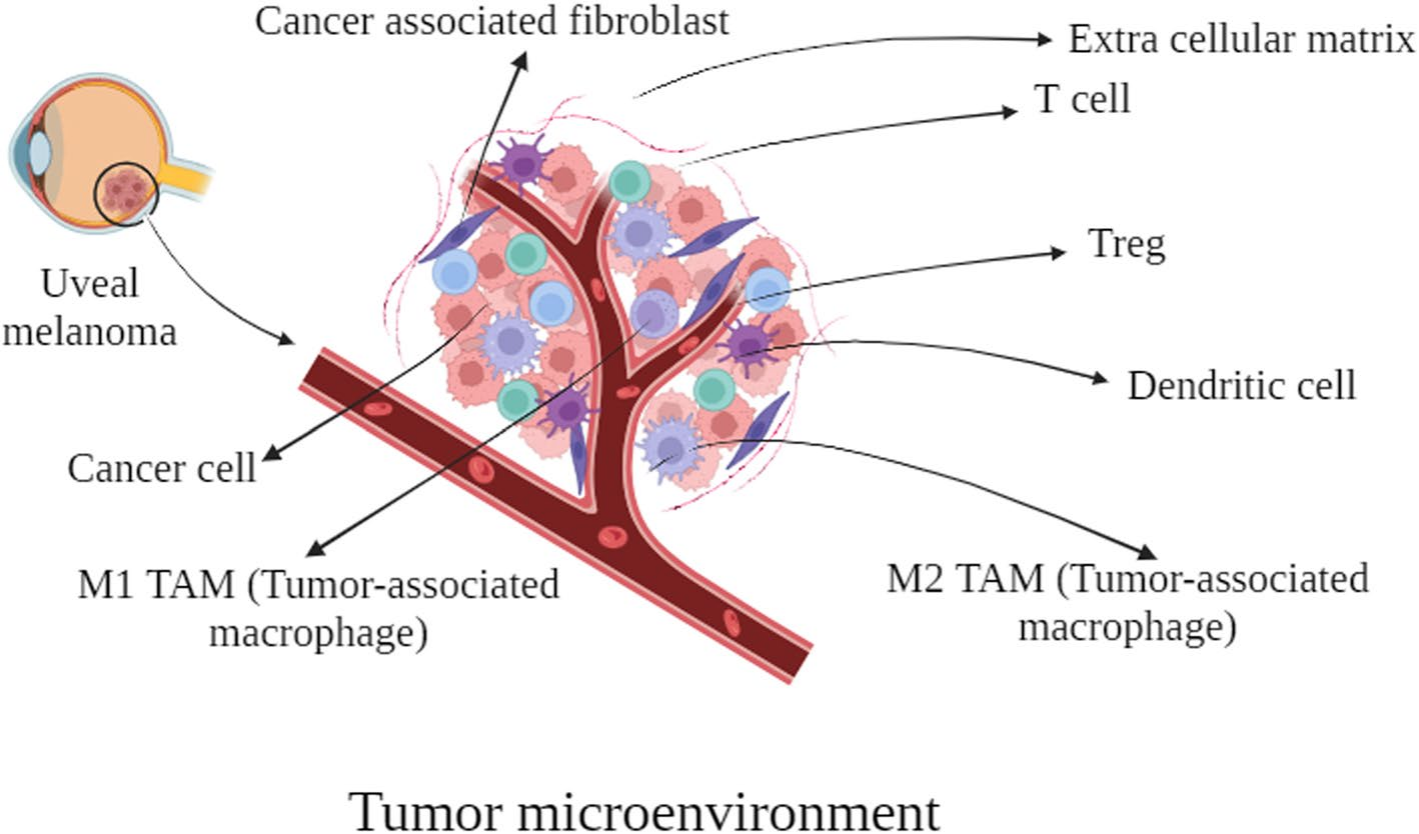
An Illustrative Depiction of the tumor microenvironment: Cancer cells within the tumor interplay with immune cells, fibroblasts, and blood vessels, shaping the complex TME. Cancer cells often modulate this environment, fostering tumor progression and metastasis. This intricate interplay is pivotal in cancer biology, as cancer cells frequently exert influence over the surrounding microenvironment, orchestrating changes that promote tumor progression and metastasis. (Created with BioRender.com)

Twenty-one types of immune cells' relative infiltration levels and expression levels of immune checkpoint genes like LAG3, CD276, HAVCR2, PDCD1, CD274, and CTLA4 have been analyzed using bioinformatic tools [132]. There are also some databases and algorithms to assess the potential responsiveness to Immune checkpoint blockers like The Cancer Immunome Atlas (TCIA) and Tumor Immune Dysfunction and Exclusion (TIDE), respectively. These studies have helped analyze differences among primary and metastatic UM and metastatic UM patients with differences in overall survival. With the help of machine learning these features and their relevant characteristics can be used for predicting new cases [133]. Du et al. have compared the primary and metastatic UM's tumor immune microenvironment and their potential response to ICB after developing a metastasis-related prognostic model [134]. Liu et al. also demonstrated the immune cell infiltrations and Immune scores for UV patients with differences in glycosylation status based on their glycosylation-related expression signature [133]. Regarding one of the important parts of TME, extracellular matrix, Li et al. have developed a prognostic model for UM based on basement membrane genes that can predict the response to immunotherapy. They also analyzed the expression patterns of these genes in different immune cells with the help of single-cell RNA sequencing (scRNA-seq) data [135]. Incidentally, High throughput gene expression analysis with bioinformatic tools paves the way for more analysis in finding different cellular patterns and expression of immunological pathways in TME that are shared by many tumors [136, 137]. Although these studies were very informative, scRNA-seq adds more information by revealing the state of every cell in the TME [138]. Figure 3 provides an illustrative depiction of the TME, showcasing the intricate interactions between cancer cells and various components such as immune cells, fibroblasts, and blood vessels. Within this dynamic ecosystem, cancer cells exert influence, often manipulating the TME to promote tumor progression and metastasis.

#### Circulating immune cells in UM

Besides the tumor microenvironment, the pattern of circulating immune cells like T cells, natural killer (NK), natural killer T (NKT), and myeloid suppressor cells and immune miRs like miR-125b, 146a, 155, 181a, and 223 have represented changes before clinical or radiographic evidence of metastasis [139]. Indeed, the soluble forms of immune checkpoints and inflammatory cytokines and chemokines were assessed for primary UM, metastatic UM, and metastatic UM undergoing anti-PD1 treatment. IL-8, HVEM levels and IDO activity showed prognostic consideration with enhancement in metastatic UM and patients with short survivals. The level of soluble immune checkpoint molecules like sCD137, sGITR, and sCD27 depicted enhancement in fast progressor metastatic UM patients compared to long survivals. Besides, sPD-1, sCD28, sCD137, sPD-L2 sLAG3, sCD80, and sTim3 also have raised in metastatic UM after ICB treatment [140].

#### Immunotherapy

Although there are no successful reports of UM immunotherapy like Immune Checkpoint Blockers, attempts are going on novel immune-based treatments. For instance, tebentafusp, an FDA-approved TCR-ScFv fusion molecule, showed overall survival benefit to UM by targeting gp100 on tumor cells and CD3 on T cells and eventually redirecting T cells toward tumor cells [128]. Accordingly, V domain Ig Suppressor T-cell Activation (VISTA) has been proposed by Salah et al. as an Immune checkpoint blockade strategy for primary UM [141]. In addition, the field of adoptive T-cell therapy is under investigation. Identifying Immunogenic neoantigens, the sequence of their cognate T Cell Receptors, and the state of these cells is critical for efficacious and sensitive treatment [142, 143]. Although the TME and intra-tumoral heterogeneity hinder this field, liquid biopsy and single-cell analysis can progress the neoantigen prediction and TCR sequencing/T cell state analysis for patients, respectively.

To sum it up, being a heterogeneous tumor in an immune-privileged and immune-suppressive organ highlights the need for a more detailed and deeper understanding, as well as real-time monitoring, of the primary and metastatic immune landscape of UVs. Accordingly, single-cell analysis of circulating cells in the blood provides a foundation for genomic and phenotypic analysis of heterogenous circulating cancer cells and immune cells.

## Implications for targeted therapy

In trying to reduce off-target effect of conventional chemotherapies, the first targeted therapies were approved to specifically target cancer cells in 1998 [144]. Small molecules and monoclonal antibodies, two main groups of targeted drugs, hinder signal transduction pathways by inhibiting protein kinase activity and ligand-receptor interactions. targeted drugs that affect proliferation, angiogenesis, metastasis, and anti-cancer immune responses include tyrosine kinase inhibitors, serine/threonine kinase inhibitors, epigenetic inhibitors, Bcl2 inhibitors, PARP inhibitors, hedgehog pathway inhibitors, proteasome inhibitors, and immune checkpoint blockers. Notwithstanding the diversity and effectiveness, intrinsic (primary) and acquired resistance to targeted therapies by diverse mechanisms is a vast challenge [145]. ITH can lead to drug resistance directly or indirectly by changing the TME dynamically [146].

Hence, according to Marusyk et al. strategies for treating high ITH tumors should do their best on ITH tumors ( by considering tumor dependencies) and affect ITH itself [53]. The targets in the former are the basic mechanism of tumors and in the latter are all sources of heterogeneity rather than genomic instability.

### Overview of targeted therapies available for melanoma

Clinical trials on targeted therapy for UM encompassing MEK inhibitors, PI3K/AKT/MTOR inhibitors, HDAC inhibitors, bromodomain and extraterminal (BET) protein family inhibitors, Tyrosine kinase receptor MET, and Multi-tyrosine kinase inhibitors. Pathways downstream driver mutations, epigenetic mechanisms and transcription are targeted by these therapies [147].

Targeting ubiquitin–proteasome system (UPS), contributing to UM malignancy, has been proposed on different enzymes involved in this system. The results of studies focused on Targeting Proteasome and E3 ubiquitin ligases like MDM2 and SKP2 directly or indirectly (upstream regulation) using small molecules and transducible peptides showed positive results, leading to some clinical trials. There are also attempts to combine UPS-targeted therapies with other targeted therapies like PKC inhibitors and chemotherapy [148].

#### Liquid biopsy for early detection

The identification of tumor-derived material in the bloodstream, commonly referred to as "liquid biopsies," has garnered significant attention in the scientific community [149]. Emerging research highlights liquid biopsies as minimally invasive tools for diagnosing, prognosing, and monitoring cancer. In uveal melanoma, advanced technologies and clinical studies have extensively explored circulating biomarkers like CTCs, ctDNA, and circulating micro-RNA (miRNA), offering promise for valuable insights and active investigation [149–155].

There is a critical need for improving the detection of minimal residual disease (MRD) among uveal melanoma patients, mainly due to the high incidence of metastatic disease. One promising method is to use of liquid biopsy, which involves analyzing rare circulating tumor cells or DNA obtained from blood samples taken from the patient's peripheral blood. Liquid biopsies are a type of test that is minimally invasive and easy to access. They help to measure the presence of micro-metastatic disease, track how the disease is progressing, and provide real-time genomic assessments of primary tumors and metastatic lesions. In uveal melanoma, which typically spreads through the bloodstream, circulating tumor cells are of particular interest. In preclinical mouse xenograft models, CTCs have been shown to initiate metastasis. Prognostic applications of CTCs have been studied in various types of solid organ malignancies, including breast, prostate, colon, bladder, and esophageal cancer. In these cases, an increase in CTC count is associated with a higher risk of metastasis [156, 157]. UM research predominantly centers on the primary tumor, even though metastases are the primary cause of patient mortality. The intricate process of tumor metastasis involves multiple complex steps, making it a challenging area of study. Liver resection is a treatment method used for UM metastases. However, it's a rare procedure and diagnostic biopsies often don't provide enough material for further research. Samples obtained from metastatic UM patients often comprise a mixture of UM cells, reactive cells, and hepatocytes, posing challenges in accurately describing the genomic profile of UM metastases. It is noteworthy that despite successful treatment of the primary tumor, some UM patients still develop metastases, implying potential dissemination of UM cells into the bloodstream before the primary tumor diagnosis and treatment. Prior investigations suggest that primary UM can metastasize before treatment initiation, leading to detectable CTCs at the time of diagnosis. However, CTCs are predominantly observed in the blood of patients with metastatic UM, while those with primary UM often exhibit no detectable CTCs. The absence of CTCs in primary UM raises uncertainties regarding whether it reflects their low numbers at diagnosis, or if CTC seeding occurs after the formation of metastatic lesions. Isolating these rare CTCs both at diagnosis and during the metastatic phase could significantly advance our understanding of the UM metastatic cascade. In essence, UM research predominantly concentrates on the primary tumor, despite metastases being the primary cause of mortality. Challenges persist in acquiring adequate research material from metastatic sites due to the intricate cellular mixture present. An improved comprehension of the presence and behavior of CTCs, along with the analysis of ctDNA, holds promise for enhancing our understanding of UM metastasis and facilitating the development of enhanced diagnostic and therapeutic approaches [158–160].

#### CTC analysis comparison with cfDNA

Generally, CTCs offer several advantages over cfDNA in cancer research and clinical practice. Firstly, CTCs hold promise in identifying both novel targets and the frequency of multiple known targets, particularly in large-scale multi-institutional cohort studies. While cfDNA may be useful for detecting defined targets in clinical trials, CTCs are preferred when resistance emerges, serving as a functional assay to guide therapeutic decisions. Moreover, CTCs provide valuable insights into cancer biology and treatment response. They can be classified based on intrinsic subtypes, allowing for a more nuanced understanding of tumor heterogeneity. Additionally, CTCs exhibit dynamic changes in epithelial-mesenchymal transition (EMT) composition, which can inform disease progression and metastasis. Despite the challenges of single-cell or low-cell number sequencing, deep sequencing of CTCs has revealed matching mutations with tumor subclones, highlighting their potential to tumor heterogeneity. Importantly, intact CTCs may represent resistant clones that are not detectable in cfDNA, making their isolation and characterization crucial for therapeutic decision-making. They can be cultured to evaluate drug resistance either in vitro or in vivo, providing valuable insights into treatment efficacy and guiding personalized therapeutic strategies. By harnessing the unique advantages of CTC analysis, researchers and clinicians can make informed decisions that optimize patient care and improve clinical outcomes. The utilization of CTCs offers distinct advantages in cancer research and clinical practice. One significant advantage lies in the potential of intact CTCs to represent resistant clones that may not be detected through ctDNA analysis. This discrepancy arises because the DNA from resistant clones may not be present in ctDNA due to various factors such as shedding dynamics or degradation. Consequently, the isolation and characterization of intact CTCs can provide valuable information regarding therapeutic decisions, including the identification of resistance mechanisms and the selection of appropriate treatment strategies. Furthermore, CTCs serve as versatile tools for functional assays, enabling the assessment of various molecular components crucial for cancer progression and treatment response. Through analysis at the DNA, RNA, and protein levels, CTCs offer insights into the dynamic molecular processes occurring within tumors. These functional assays not only enhance our understanding of tumor biology but also facilitate the evaluation of potential therapeutic targets and the prediction of treatment outcomes [161, 162].

A comprehensive histopathological analysis of 643 eyes from UM patients unveiled a high incidence of intravascular tumor growth, along with other unfavorable prognostic factors such as epithelioid cell type, intrascleral growth, and large tumor size. Given the limited lymphatic drainage in the eye, the dissemination of CTCs through the bloodstream is hypothesized to be a crucial step in the metastatic cascade [10, 11]. Multiple studies across various cancer types have demonstrated the potential of CTCs as prognostic markers, providing insights into prognosis, therapy response, and disease recurrence. In the medical field, the presence of the AR-V7 variant in CTCs has been helpful for doctors in treating castrate-resistant prostate cancer patients, resulting in better outcomes than current standards. Similarly, CTC enumeration has shown prognostic value in non-metastatic breast cancer, with CTCs detected before neoadjuvant chemotherapy being associated with decreased overall and disease-free survival. CTCs have also been used for staging metastatic breast cancer. In cutaneous melanoma, the detection of CTCs has been linked to relapse-free survival, making them potential biomarkers to identify patients who may benefit from adjuvant therapies. Additionally, studies have revealed that cutaneous melanoma CTCs exhibit a heterogeneous nature, respond dynamically to therapy, and have specific CTC phenotypes with predictive value in terms of response and progression-free survival, highlighting their importance as cancer biomarkers [4, 5, 21, 163–168].

In the realm of uveal melanoma, initially spotting CTCs depended on cytochemistry and conventional microscopy techniques. Reverse transcriptase-polymerase chain reaction (RT-PCR) was commonly utilized to pinpoint UM-specific mRNA transcripts, primarily focusing on indicators such as tyrosinase, melanoma antigen recognized by T-cells 1 (MART1), glycoprotein 100 (gp100), and epithelial to mesenchymal transition markers. Although RT-PCR-based investigations into gene expression typically uncovered poorer prognoses in primary UM cases, the incidence of patients with detectable CTCs remained relatively limited, with certain studies unable to detect CTCs in primary UM [19, 157, 164, 169–175]. Nevertheless, RT-PCR by itself does not offer conclusive proof of CTC presence, nor does it furnish phenotypic or genotypic insights, nor differentiate between CTCs and circulating RNA. Hence, the direct capture and enumeration of CTCs have been investigated as prognostic biomarkers in patients with uveal melanoma. Immunomagnetic isolation, employing antibodies linked to ferric fluids or beads that target prevalent UM surface antigens, has emerged as the primary technique for enriching CTCs [167, 176–178]. Melanoma-associated chondroitin sulfate proteoglycan (MCSP) has frequently been employed as a target for capturing UM CTCs, with detection rates ranging from 1.6% to 19% in primary UM cases, correlating with poor prognostic features in some studies. However, other investigations did not find a correlation between CTC quantity and metastatic potential. Other surface antigens, such as melanoma cell adhesion molecule (MCAM), have also been utilized for immunomagnetic isolation using the CellSearch system, showing capture rates ranging from 30 to 50% in primary UM cases. Enumeration of CTCs in early-stage UM has demonstrated promise in predicting increased metastatic risk and mortality. Multimarker approaches, targeting multiple surface antigens like CD63 and gp100, have achieved high detection rates exceeding 90%. Additionally, filtration techniques have been employed for CTC isolation in primary UM, with detection rates ranging from 31 to 54% in localized disease cases. The presence of more than 10 CTCs per 10 mL of blood using the ISET filtration system has been associated with poorer prognosis over 24 months [18, 21, 158, 159, 179–186].

While most studies on CTC isolation have focused on primary UM, investigations have also explored metastatic UM. The CellSearch system has been utilized for detecting CTCs in metastatic UM, with their presence strongly associated with military hepatic metastases, overall tumor burden, progression-free survival, and overall survival. Comparisons between arterial and venous blood have revealed higher CTC counts in arterial blood, indicating potential clinical significance. Recent studies have reported higher percentages of metastatic patients with detectable CTCs compared to those at the primary localized stage.

Although there are different methods used to detect circulating tumor cells, there is inconsistent evidence about whether counting them is a useful way to predict outcomes in people with metastatic uveal melanoma (UM). However, the available data does suggest that CTCs are present in the blood of UM patients who have the primary disease.

Numerous factors can impact the retrieval and identification of CTCs, extending beyond the selection of specific detection methodologies. For instance, the utilization of fixative tubes like Streck or CellSave has shown notable enhancements in CTC retrieval when analysis is delayed. Nevertheless, EDTA is generally deemed suitable for processing if conducted promptly following blood collection. In studies concerning UM, the CellSearch method has commonly employed companion CellSave tubes, while others have utilized EDTA or heparin. In vitro examinations involving spiked "CTC mimic" in EDTA tubes revealed a marked reduction in the number of retrieved cells after one hour. However, it's crucial to note that the study didn't specify the storage temperature, which might also be a pivotal factor, akin to circulating tumor DNA. Additional critical factors encompass the type of input sample, whether whole blood (ISET/CellSearch), white blood cells post-peripheral blood mononuclear cell separation, or red blood cell lysis. These variations in sample type can potentially introduce confounding variables during isolation and analysis due to fluctuations in background levels and types of leukocytes, historically presenting inconsistency in UM research [186–188].

#### Precision medicine and personalized treatment approaches

Precision medicine, a population-based approach to finding out the molecular mechanism underlying disease, relies on omics data (bulk and single-cell level) and precedes targets and biomarker discovery for subsequent uses in Personalized medicine. In Personalized medicine, special treatment is proposed for patients based on their data. Liquid biopsy (CTC), a non-invasive and real-time tool for biomarker detection, helps in gathering personalized level data from patients [189, 190]. Single-cell analysis as a high-resolution heterogeneity analysis accompanied by adoptive personalized medicine can take part in facing drug resistance [191, 192].

Precision medicine resulted in better clinical outcomes, including longer Progression-free survival and overall survival [97, 193]. The higher matching score, Targeting more alterations, correlates with better clinical results and highlights the need for combinations of agents to maximize effectiveness [192, 194].

Leyvraz et al. have conducted Treat20 Plus, a personalized precision medicine based on molecular analysis (WGS, WES, RNA-seq). Although overall survival showed no significant differences between patients with genome-matched treatment and unmatched treatment, it was in line with other therapies like chemotherapy or checkpoint inhibitors [42].

Performing invasive tests to monitor the dynamics of lung cancer can be challenging and may not reflect the current tumor dynamics and drug sensitivity, which can change during therapy. Therefore, developing a noninvasive biomarker to monitor the dynamics of lung cancer in real time is crucial. This "liquid biopsy" can be an ideal therapeutic strategy for individual cancer patients. It can facilitate the development of tailor-made cancer management programs [190, 192, 195]. Although CTCs originate from the primary tumor, they are distinct from primary tumor cells [6], EMT transition properties aid cancer cells in breaking free from the primary tumor, allowing them to enter the bloodstream and spread in clusters as CTCs, thereby increasing their potential to metastasize. Additionally, these cancer cells exhibit stemness features that enhance their ability to initiate metastasis. Recently, the concept of MRD has been introduced to ocular oncology for vitreoretinal lymphoma by Stacey and Pulido [190, 195].

Unraveling Phenotype switching [52], a prominent heterogeneous way for drug resistance can improve Personalized medicine. So, single-cell multi-omics analysis of CTC can help reach this goal [190, 192, 195].

### Challenges and limitations of targeted therapy in melanoma

The utilization of tissue biopsy in uveal melanoma remains contentious because of the potential risk of extraocular dissemination. As a result, there is a pressing necessity to pinpoint dependable and non-invasive biomarkers for identifying patients at elevated risk of metastasis.

#### Implication of single-cell in UM

Even though single-cell-based technologies are highlighted in many studies, the ongoing field of bulk RNA seq analysis with the help of emerging bioinformatic tools is undeniable. These two fields are complementary with their specific advantages and limitations. In comparison to single-cell RNA seq, Bulk RNA seq data harbor fewer noises [133, 178, 196, 197]. Also, the relative abundance of cell types that are obtained with single cells of solid tissue is a biased duo to the cell dissociation step [197, 198]. However, Low resolution [197], and the inability to analyze cell type-specific expression patterns can limit deconvolution results [199, 200]. Additionally, deconvolution methods which are inexpensive methods for analyzing cell type abundances, are themselves dependent on a reference single-cell RNA seq [200]. Single-cell isolation techniques have emerged as powerful tools in diverse research fields, particularly in the investigation of complex biological systems like cancer. These techniques enable precise and efficient isolation of individual cells from heterogeneous populations, facilitating in-depth analysis of their unique genomic, transcriptomic, proteomic, and metabolomic characteristics. Several methodologies, including fluorescence-activated cell sorting (FACS), microfluidics-based systems, laser capture microdissection (LCM), and droplet-based technologies, have been developed to facilitate single-cell isolation. By allowing the examination of cellular heterogeneity at the individual cell level, these techniques provide valuable insights into cellular diversity, clonal evolution, cellular interactions, and disease mechanisms. Consequently, they advance our understanding of complex biological processes and drive the development of targeted therapies and personalized medicine approaches [195, 201–205].

Single-cell isolation methods are indispensable for extracting homogeneous clusters of tumor cells from the surrounding cellular environment, which may comprise infiltrating immune cells, normal cells, or rare cell subsets constituting less than 1% of the overall cell population. While techniques such as flow cytometry, microfluidics platforms, and manual micromanipulation can effectively isolate abundant tumor cell populations, identifying and isolating rare cancer cells poses considerable obstacles. Nonetheless, there is a range of methodologies and platforms currently accessible for single-cell isolation, rendering them particularly beneficial for detecting CTCs. The detection of CTCs has garnered attention due to the feasibility of liquid biopsies, like blood samples, and the observed correlation between heightened CTC counts and an amplified risk of metastasis in breast and other cancers. However, the scarcity of CTCs in the bloodstream presents a significant challenge to their detection [206–208]. To improve the detection of CTCs, various enrichment protocols have been developed. These include ficoll density gradient separation, erythrocytes, and immunomagnetic selection systems using Dynabeads or Miltenyi CD45 beads, which can be combined with other single-cell isolation techniques. Certain platforms, such as CellSearch and Magsweeper, have been specifically designed to identify CTCs. These systems incorporate cytokeratin markers to enhance CTC detection. Currently, CellSearch is the only system approved by the Food and Drug Administration for clinical use in detecting CTCs in breast, colorectal, and prostate cancers [156, 209, 210].

Several studies have reported that it may be possible to determine a patient's risk of developing metastatic disease by detecting either CTCs or ctDNA in their blood samples in cases of uveal melanoma. However, currently, the sensitivity and specificity of these tests are low. Additionally, some studies have found that a longer time between diagnosis and treatment of uveal melanoma is associated with a higher risk of metastatic death.

In recent years, single-cell analysis has emerged as a powerful approach for unraveling the complex biology of tumors, including uveal melanoma, and has shown great promise in elucidating the intricacies of this disease, as well as in the detection and characterization of CTCs [211–213]. The conventional bulk analysis techniques often fail to capture the cellular heterogeneity and dynamic interplay between different cell populations in uveal melanoma. In contrast, single-cell analysis techniques, such as scRNA-seq, offer unprecedented opportunities to study individual tumor cells, enabling the identification and characterization of distinct cellular subpopulations and their contributions to disease progression and treatment resistance. By profiling the transcriptomes of individual cells, scRNA-seq has revealed diverse subpopulations within uveal melanoma, shedding light on the tumor microenvironment, immune cell infiltration, gene expression changes associated with metastasis, drug resistance, and potential therapeutic targets [176, 214–216]. Detection and characterization of CTCs provide valuable insights into the biology of metastatic disease and serve as a minimally invasive tool for monitoring disease progression and treatment response [159, 180]. Single-cell analysis techniques have revolutionized the study of CTCs by enabling the analysis of individual CTCs, their genetic profiles, and expression signatures. In the context of uveal melanoma, single-cell genomics, and transcriptomics have led to significant advancements in understanding the heterogeneity of CTCs, identifying unique gene expression patterns, genomic alterations, and potential drivers of metastasis [185, 217, 218]. One such technique, single-cell RNA sequencing, enables the high-resolution analysis of transcriptomic profiles in individual cells. This approach has proven instrumental in unraveling cellular behavior and interactions within the TME, facilitating a deeper understanding of cancer biology. By applying scRNA-seq, researchers have made significant strides in characterizing the TME and identifying tumor-infiltrating immune cells that play critical roles in tumor survival in breast and pancreatic carcinomas. These findings have enhanced our knowledge of cancer behavior and the development of resistance mechanisms [15, 210, 218]. Single-cell RNA sequencing enables the cytological characterization of CTCs obtained from lung cancer patients, based on the conventional morphological criteria employed by cytopathologists to differentiate between benign and malignant epithelial cells [18, 19, 115].

**Fig. 4:**
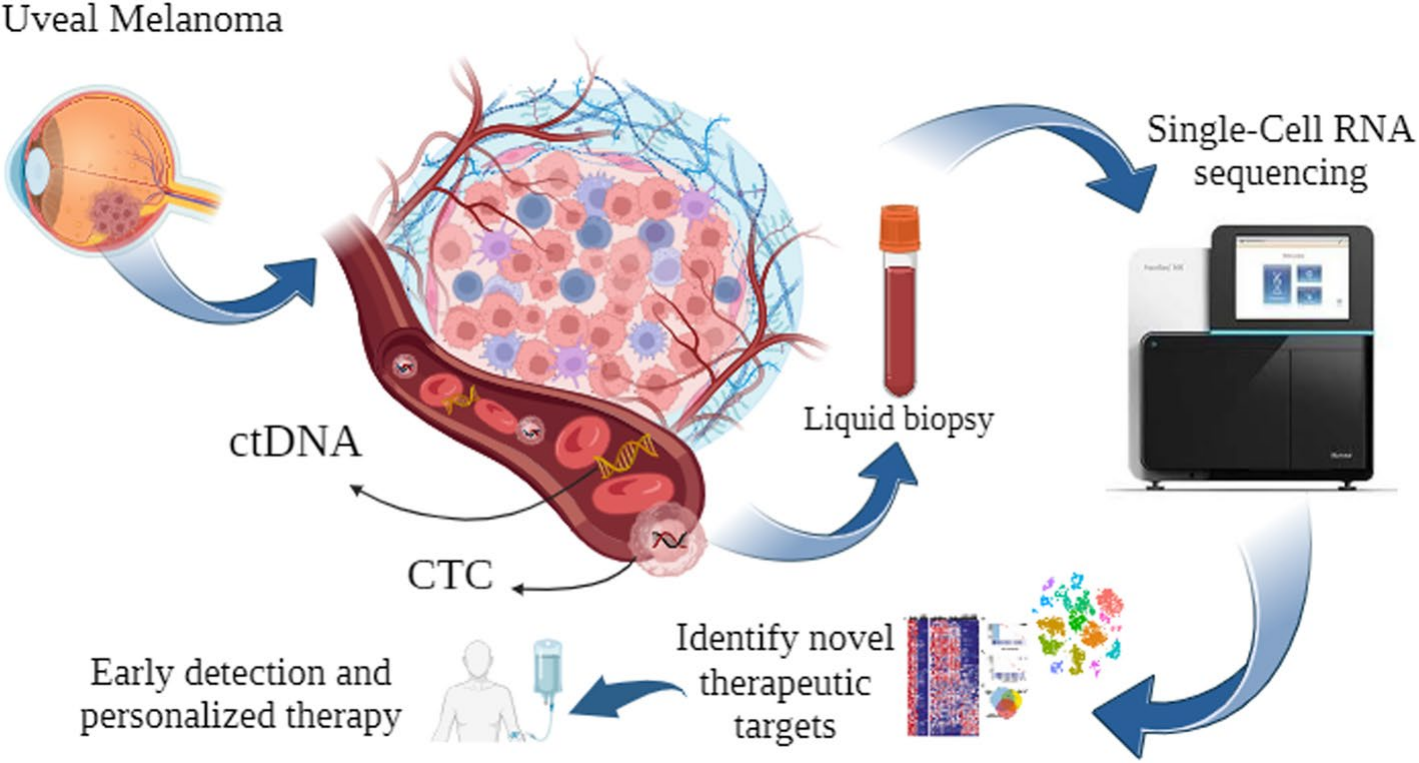
Schematic Overview of the Integration of CTCs through Liquid Biopsy and Single-cell Analysis as a Promising Therapeutic Approach for Uveal Melanoma. This figure illustrates the integration of CTCs via liquid biopsy and single-cell analysis as a promising therapeutic strategy for uveal melanoma. It demonstrates the use of non-invasive liquid biopsy to capture CTCs released from the primary tumor into the bloodstream. Following this, single-cell analysis techniques are employed to characterize individual CTCs, revealing crucial genetic mutations and signaling pathways associated with uveal melanoma progression. (Created with BioRender.com)

Techniques such as single-cell DNA sequencing and array-based CNV analysis, coupled with whole-genome amplification, enable the assessment of mutational profiles at the single-cell level. These approaches have been successfully employed to identify resistant subclones and delineate mutational landscapes in acute lymphoblastic leukemia, colon cancer, and breast cancer. Such advancements contribute to improved prognostication and provide valuable information for personalized treatment strategies [195, 219–222]. Overall, single-cell transcriptomics and genomics have revolutionized our ability to study individual cells, unravel cellular heterogeneity, and uncover critical molecular events that drive cancer progression and therapy resistance [195, 223, 224]. The advent of single-cell next-generation sequencing techniques has revolutionized the analysis of DNA, RNA, proteins, and metabolites at the cellular level, thus making significant contributions to the field of oncological theragnostic. Single-cell transcriptome analysis revealed heterogeneity in the CTC subpopulation. Pre-processing procedures for single-cell RNA sequencing include read alignment and quantification, quality control, normalization and batch correction, dimensionality reduction, and cell annotation [195, 225–230]. Figure 4 presents a schematic overview of the integration of CTCs through liquid biopsy and single-cell analysis as a promising therapeutic approach for uveal melanoma. This visualization highlights the potential of harnessing CTCs for personalized treatment strategies, offering insights into tumor heterogeneity and aiding in the development of targeted therapies for this challenging disease.

#### Advances in single-cell technologies for uveal melanoma

Emphasizing single-cell transcriptomics and genomics, researchers strive to characterize the diverse cell populations within UM tumors, encompassing melanoma cells, infiltrating immune cells, and stromal cells. These cutting-edge techniques offer detailed transcriptomic profiles, unveiling insights into cellular functions, interactions, and the TME of UM. Additionally, single-cell DNA sequencing allows for the identification of driver mutations and genomic alterations associated with UM progression and metastasis. Moreover, investigators are harnessing single-cell technologies to investigate circulating tumor cells in the bloodstream, which serve as valuable biomarkers for assessing metastatic risk and treatment response. By integrating multi-omics data derived from single-cell analyses, novel therapeutic targets can be discovered, paving the way for personalized treatment strategies tailored to UM patients [46, 195, 231–233].

**Fig. 5:**
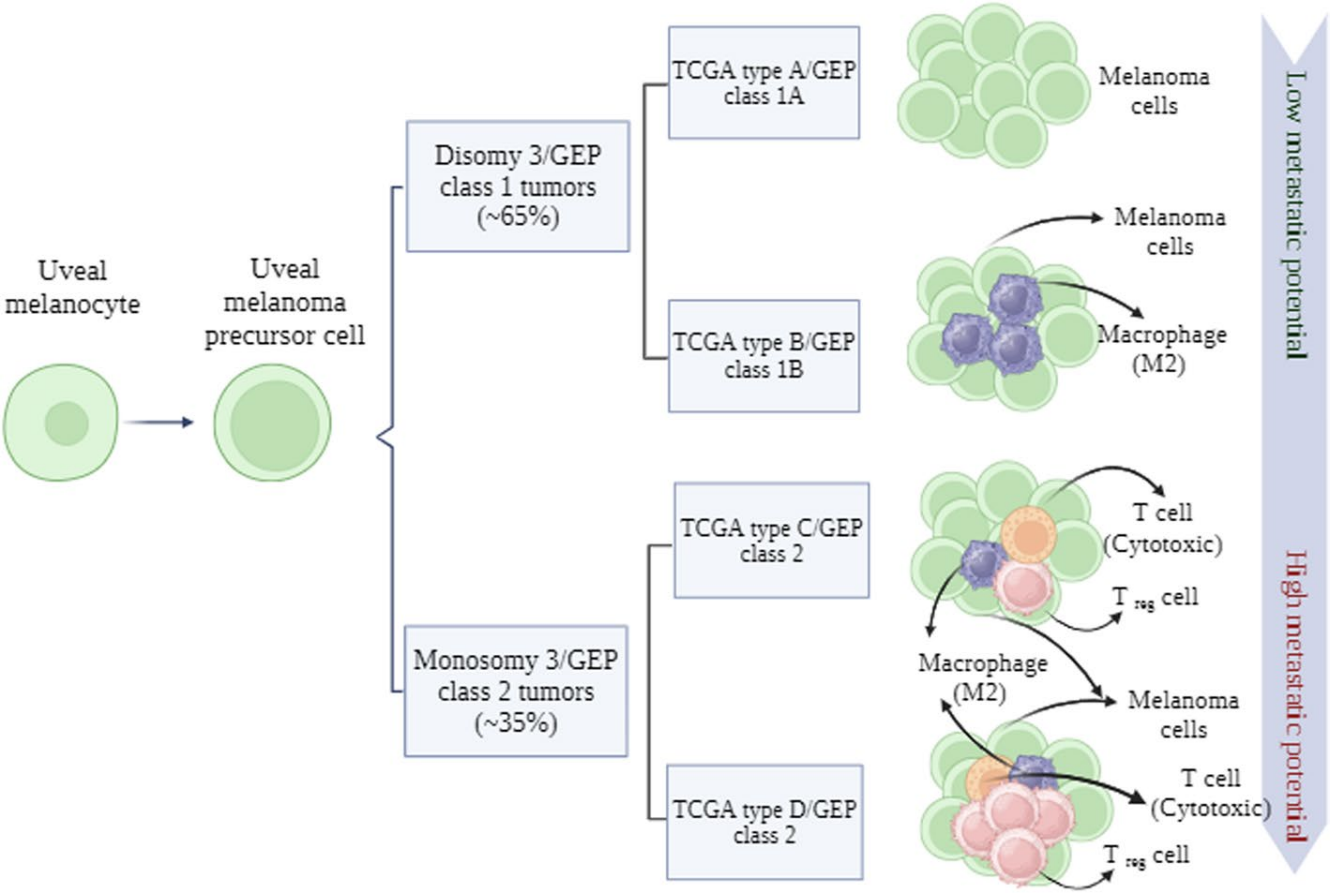
The Progression from Uveal Melanocyte to Melanoma: Insights into the Evolutionary Process. This figure encapsulates a visual depiction of the evolutionary journey from normal uveal melanocytes to malignant melanoma cells within the context of uveal melanoma. It elucidates the key genetic mutations that drive this progression, shedding light on the complexities of tumor growth. (Created with BioRender.com)

Uveal melanoma's tumor microenvironment encompasses a diverse array of cell populations, including melanoma cells, infiltrating immune cells like macrophages and lymphocytes, stromal cells, and blood vessels. Extensive research has been conducted on key mutations in UM, such as GNA1 or GNA11, EIF1AX, SF3B1, and BAP1, elucidating their association with varied prognoses and metastatic risk. Techniques like microdissection and NGS have contributed to the understanding of the TME; however, the majority of studies have been confined to bulk cell analysis. Recent investigations have delved deeper into UM, encompassing whole-genome analysis that identified pivotal events such as BAP1 mutations, as well as novel mutations involving epigenetic regulators and deletions of CDKN2a associated with metastatic tumors. Transcriptomic profiling of liver samples has unveiled the contribution of M2 macrophages and revealed genes linked to immunosuppressive environments and UM. While single-cell approaches in UM are emerging, the existing literature on this subject remains limited, with a predominant focus on circulating tumor cells [6, 23, 70, 195, 234–239]. In Fig. 5, we present an overview of the evolutionary process from uveal melanocyte to melanoma, highlighting the pertinent genetic factors involved in this intricate disease pathway.

By scrutinizing individual cells within the tumor and its neighboring milieu, researchers can unravel the dynamics and interactions of various cell populations, encompassing melanoma cells, immune cells, stromal cells, and blood vessels. Single-cell transcriptomics has facilitated the characterization of gene expression patterns at a cellular level, unveiling the heterogeneity and functional diversity inherent in the UM microenvironment. This breakthrough has led to the identification of specific subsets of immune cells and their activation states, furnishing pivotal insights into the immune response against the tumor. Furthermore, single-cell genomics has enabled the detection of genetic alterations and driver mutations at the level of individual cells, thereby enhancing our comprehension of the genomic landscape and clonal evolution within UM. By unraveling the cellular constituents and molecular attributes of the UM microenvironment, single-cell approaches hold tremendous potential for uncovering novel therapeutic targets and propelling personalized treatment strategies for this aggressive ocular malignancy [61, 184].

In a recent investigation, scientists explored the microenvironment of UM using a single-cell approach. They isolated 59,915 individual cells from both tumor and non-neoplastic tissues of eight primary and three metastatic UM samples and conducted single-cell RNA sequencing using the 10 × Genomics platform. The gene expression profiles derived from the single-cell RNA sequencing data exhibited clustering patterns similar to those seen in Class 1 and Class 2 GEP clinical prognostic tests for UM. However, at the single-cell level, the expression of specific genes within these tests (such as EIF1B, HTR2B, ECM1, CDH1, ROBO1, and SATB1) was primarily observed in tumor cells and T cells, respectively. Moreover, the single-cell CNV analysis provided evidence indicating that canonical CNVs do not always occur as a single early event but continue to evolve alongside tumor progression. Class 1 UM cases displayed losses of 1p, 3, and 8p, while Class 2 UM cases exhibited gains of 6p and 6q. Furthermore, the study unveiled the expression of the checkpoint marker LAG3 in tumor-infiltrating immune cells, suggesting its potential as a candidate for immune checkpoint blockade in high-risk UM patients, who typically demonstrate poor response to PD1 and CTLA4 checkpoint inhibitors. This investigation illustrates how single-cell analysis offers a higher resolution of transcriptomic alterations in individual UM and tumor-infiltrating immune cells, thereby corroborating findings from bulk-cell approaches. A previous study conducted a thorough analysis of single-cell transcriptomes from 11 UM patients, revealing the presence of four distinct macrophage subsets. Among these subsets, MΦ-C4 emerged with distinct characteristics, exhibiting low expression of M1 and M2 signature genes, along with a significant loss of inflammatory pathways and heightened signaling related to proliferation, mitochondrial functions, and metabolism. Notably, the increased infiltration of the MΦ-C4 subset correlated with aggressive tumor behavior and poor prognosis in UM patients, suggesting its potential as an independent prognostic indicator. In response to these insights, the study proposed an innovative subtyping scheme based on macrophages, which integrated transcriptional signatures of MΦ-C4 alongside machine learning techniques. This scheme effectively stratified patients into MΦ-C4-enriched or MΦ-C4-depleted subtypes, showcasing distinct clinical outcomes. Additionally, the study emphasized the therapeutic promise of targeting macrophage subsets, particularly MΦ-C4, as a compelling strategy to mitigate the progression of metastatic disease and enhance patient outcomes in UM. In another investigation, Single-cell DNA sequencing was conducted utilizing the Chromium instrument, facilitating the analysis of genomic DNA at the individual cell level. This comprehensive sequencing approach yielded insights into clonal expansion and genomic aberrations within UM cells. Notably, the data unveiled subclonal genomic complexities and delineated specific genomic aberrations linked to the co-evolution of UM cells alongside immune cells. By scrutinizing the single-cell DNA sequencing data, researchers gained a deeper understanding of the evolutionary trajectories of UM cells and their intricate interplay within the tumor microenvironment. Furthermore, this analysis provided invaluable insights into the genomic heterogeneity and transcriptional profiles of UM cells. Moreover, Single-cell DNA sequencing proved instrumental in identifying clonally expanded T cells and plasma cells within the tumor microenvironment, shedding light on the presence of immune responses and antibody-mediated immunity in UM. This sophisticated sequencing technique thus offers a nuanced perspective on the genomic landscape of UM and the dynamic interactions occurring within the tumor microenvironment, laying the groundwork for further elucidating the mechanisms underlying UM progression and immune evasion strategies [6, 58, 61, 240]. In another recent study, novel expressions of long non-coding RNAs (lncRNAs) in UM were identified and found to be associated with BAP1 functionality. The study discovered 104 novel transcripts, with 32 showing differential expression between Class 1 and Class 2 UMs. Subsequently, single-cell RNA sequencing revealed 10 leading lncRNAs that were exclusively expressed in tumor cells, highlighting the potential of single-cell approaches for further exploration of UM [64, 195, 241].

#### Advancing the detection and analysis of CTCs in UM through single-cell approaches

Microfluidics technology has been effective in capturing and enumerating CTCs, providing important prognostic information. Furthermore, scRNA-seq has enabled the molecular profiling of UM CTCs, revealing gene expression patterns associated with metastasis and therapeutic resistance. These single-cell approaches present unique opportunities to study the dynamics and molecular characteristics of CTCs in UM, potentially guiding the development of targeted therapies and improved prognostic tools for this aggressive ocular cancer [157, 195, 242–245].

Certain markers are used to detect CTCs in melanoma patients. These markers include melanoma-associated chondroitin sulfate proteoglycan, melanoma cell adhesion molecule, NKI/beteb, NKI/C3, and high-molecular-weight melanoma-associated antigen (MHW-MAA). Using a combination of two antibodies is more effective in isolating CTCs in uveal melanoma. The widely used CellSearch technique involves immunomagnetic enrichment of CD146 melanoma cells followed by staining with MHW-MAA. Studies have demonstrated a correlation between the presence of CTCs in UM and the extent of hepatic metastasis and overall survival, although contradictory findings exist. Examining CTCs through single-cell analyses in patients with nevi and non-metastatic UM compared to those with metastasis could provide insights into their clinical significance. Previous studies have employed real-time PCR to detect biomarkers like tyrosinase messenger RNA and MelanA/MART1 in CTCs. With the advent of single-cell isolation and analysis techniques, novel biomarkers and their expression levels can be determined, providing further understanding of their metastatic relevance. Tissue biopsies face limitations due to intra-tumoral heterogeneity, making liquid biopsies, including CTC analysis, circulating tumor DNA, and circulating microRNA, more informative. While current studies on CTC detection in UM may present inconsistencies, the application of single-cell RNA and DNA analysis on CTCs represents a promising avenue for future applications of single-cell technology in liquid biopsies [159, 174, 176, 183–185, 195, 246–248].

UM is surgically excised and then submerged in saltwater for transportation. The tissue is then subjected to enzymatic lysis to degrade individual cells and segregate them into distinct micro-environments. Fluid partitioning is used to release mRNA, which is then converted into cDNA by reverse transcription and amplified afterward. Following the sequencing of the created cDNA libraries, the sequenced reads are aligned to the human genome using a variety of computer methods. Following quality assurance procedures, the reads are evaluated to confirm the fraction of mitochondrial DNA, ensuring that only whole cells are included in the study. Next, an expression matrix that shows the number of reads per gene per cell is derived. After the data has been normalized and dimensionality reduction techniques have been used, transcriptionally similar cells are clustered together. The development of cells over time is investigated using a technique called trajectory analysis. Different approaches, such as heatmaps, dot plots, or violin plots, can be used to depict cell differential gene expression.

In solid cancer, ITH presents itself as an inherent challenge. The utilization of single-cell analyses enables the identification of distinct cell types comprising a tumor, facilitating the examination of their interactions within the TME. Through such analyses, one can illustrate the dynamic changes occurring during tumor development and in response to external factors like therapeutic interventions. Understanding the biology of coexisting cell states within the tumor and their respective markers is crucial for predicting the evolutionary trajectory of UM. Moreover, these analyses not only shed light on ITH and the various transcriptional cell states present in UMs but also unveil the complexities of their microenvironment. An intriguing approach to comprehending ITH and its role in therapeutic resistance and cancer recurrence involves analyzing CTCs and cfDNA. Genome-wide single-cell RNA sequencing and DNA sequencing techniques conducted on CTCs have provided valuable insights into tumor heterogeneity [195, 249, 250].

Overall, when conducting high-throughput sequencing on tumor tissue, millions of cells are examined together as a composite sample. While this method offers a broad perspective on the genomic features of the cells, it overlooks the heterogeneity inherent in tumor cells. As a result, genetic material from crucial cells like cancer stem cells (CSCs), CTCs, and other functionally significant yet low-abundance cells may be diluted. Fortunately, the emergence of single-cell sequencing technology has effectively remedied this limitation [156, 192, 195, 251].

Single-cell sequencing analysis of CTCs enables a comprehensive exploration of differences in single-cell genomes, transcriptomes, and epigenetic profiles across peripheral blood CTCs, primary tumor sites, metastatic lesions, and involved lymph nodes. This methodology mitigates the impact of tumor heterogeneity, offering a fresh perspective for understanding the fundamental biological processes driving tumor onset and advancement. Its utility extends to a range of cancer types, encompassing breast cancer, colorectal cancer, malignant melanoma, lung cancer, and prostate cancer, thereby propelling advancements in cancer research efforts [192, 195, 251].

Single-cell whole-genome sequencing analysis of CTCs in the peripheral blood of patients with solid tumors serves as a crucial indicator in assessing tumor progression. This method offers valuable insights into the intricate processes of tumor evolution, heterogeneity, and resistance to drugs. The identification of gene mutations, aids in the discovery of novel driver genes and enhances our understanding of the clonal lineage and evolutionary trajectories of tumors. Furthermore, it enables the discernment of genetic sequence disparities among tumor subtypes, leading to the identification of new biomarkers.

The utilization of single-cell sequencing furnishes a diverse array of data, mitigating the constraints associated with tumor classification based solely on a single biopsy. Consequently, it proves particularly beneficial in early tumor detection, prognostication, guiding therapeutic drug selection, and monitoring disease recurrence. Of paramount importance is the noninvasive nature of tumor diagnosis and prognosis prediction facilitated by single-cell sequencing, underscoring its clinical significance.

Through the integration of single-cell isolation methods with scRNA-seq and scDNA-seq, it becomes feasible to detect and contrast individual melanoma cells circulating in the bloodstream with the primary UM clone. This approach aids in assessing the metastatic capacity of CTCs [195, 251, 252].

Furthermore, scRNA-seq examination of the TME in uveal melanoma UM has unveiled a noteworthy finding: immune cells in UM express LAG3 instead of the conventional ligands targeted in immunotherapeutic approaches. The significance of this discovery underscores the need for further investigation.

The efficacy of metastatic UM research is substantially hindered by the constraints imposed by small sample sizes, which severely diminish the study's statistical power. Moreover, the accessibility of single-cell tools remains limited across various institutions and countries [195, 252, 253].

Currently, the available prognostic tools can only identify patients at high risk necessitating heightened surveillance. There exists a notable disparity in managing metastatic uveal melanoma (MUM) owing to the absence of contemporary therapies. Nonetheless, emerging evidence indicates that analyzing CTCs and circulating free DNA, particularly in high-risk UM patients, could enhance mUM surveillance. Indeed, employing single-cell analysis to explore the tumor cell microenvironment and CTCs has already yielded fresh insights into UM tumor biology [239, 252, 253].

Previous research has utilized real-time PCR methods to illustrate that the presence of CTCs aligns with the expression of biomarkers such as tyrosinase messenger RNA and MelanA/MART1 in blood samples. With the emergence of single-cell isolation and analytical techniques, we now possess the capability to examine both the individual-cell expression patterns of these established biomarkers and explore novel biomarkers that may hold significance for metastasis. Although our ability to predict metastatic risk is relatively accurate, we still lack preclinical evidence of metastasis and encounter challenges in identifying biomarkers suitable for targeted therapy. Single-cell investigation offers a promising avenue to address these gaps in UM research. Indeed, the growing adoption of single-cell technologies facilitates the detection of circulating tumor cells and the characterization of transcriptomic profiles in individual, drug-resistant tumor cells. These advancements have facilitated the identification of viable biomarkers conducive to targeted therapeutic interventions [4, 20, 174, 253, 254].

Several challenges persist in elucidating the extent to which immunoediting influences intra-tumoral heterogeneity in untreated patients. One such challenge arises from the limitations inherent in neoantigen prediction algorithms, which may fail to accurately identify subtle signals of immunoediting within data characterized by inherent noise. Addressing this issue necessitates the utilization of single-cell analysis to comprehensively capture and analyze these nuanced alterations [174, 253].

## Future directions

Besides uncovering driver mutations and biomarkers, unraveling the consequences of these alterations can improve UM management [255]. Underscoring the immune landscape of TME, changing the TME with the help of oncolytic viruses, and efforts to redirect effector T cells toward tumor cells would pave the way for more successful treatment [124, 255, 256]. In addition, developing target-specific aptamers, new candidates of small molecules, and small interfering RNAs can empower the field of UM treatment [257, 258].

Ongoing research on UM through multi-omics data and DNA topology analysis, leverages a deeper understanding of precise mechanisms of gene expression dysregulation and tumorigenesis [259].

Although almost the entire research showed no chance of an immune checkpoint blocker for UM, it showed promising results in a minority of patients with high tumor mutational burden (TMB) [42]. It emphasizes the potential power of CTC analysis for evaluating TMB for UM.

Traditional bulk analysis techniques have been inadequate for comprehensively studying UM due to their limitations in capturing the complexity of tumor heterogeneity and cell interactions. However, the advent of single-cell analysis, particularly scRNA-seq, has transformed our understanding of UM by enabling precise profiling of individual cell transcriptomes. Additionally, the analysis of CTCs has provided valuable insights into UM progression and outcomes, with CTC presence in early-stage UM indicating a higher risk of distant metastasis and poorer prognosis.

In conclusion, the integration of CTC analysis with single-cell techniques offers great potential for advancing UM research and clinical management, promising improved risk assessment, early detection, and treatment monitoring, marking a significant step towards enhancing patient outcomes in this challenging disease. The detection and characterization of CTCs hold promise for understanding metastasis and monitoring disease progression in UM. Single-cell analysis techniques have revolutionized the study of CTCs, enabled the analysis of individual cells, and revealed their genetic profiles, gene expression patterns, and metastasis-related signatures [154, 190, 195].

## Conclusion

Unraveling the molecular landscape of UM and understanding its heterogeneity significantly enhances our comprehension of this cancer's biology. While genomic alterations and driver mutations in UM offer potential targets for therapy, challenges persist in developing effective treatments. Despite the availability of population-based multi-omics data and computational biology-driven targets, the biological significance of these targets within relevant pathways remains uncertain. Focusing on driver mutations and other alterations, alongside their associated biological pathways, holds promise for uncovering emergent upstream or downstream targets unaffected by heterogeneity. Moreover, increased emphasis on the tumor microenvironment, particularly the liver, is essential. Additionally, identifying precise metastasis-related biomarkers and testing them in all UM patients can facilitate personalized treatment approaches, minimizing adverse effects. Liquid biopsies, notably CTC and ctDNA analysis, show great potential in advancing UM management by aiding in metastatic risk assessment, enabling early interventions, and guiding patient selection for clinical trials. Furthermore, ctDNA testing offers non-invasive early detection and treatment monitoring, potentially predicting responses to immune checkpoint blockade therapy. Advancements in single-cell technologies provide molecular insights into UM and potential therapeutic targets, although challenges such as standardization and the need for larger trials persist due to UM's rarity. Innovative tissue analysis methods such as single-cell analysis offer promise for the future, providing valuable insights into CTC characterization and the tumor microenvironment, thereby identifying novel therapeutic targets and biomarkers essential for more effective treatments. Overall, integrating these approaches into UM research promises transformative insights and improved diagnostic and therapeutic strategies in the coming decade.

## Data Availability

Not applicable.

## Abbreviations

BET: Bromodomain and extra terminal
BAP1: BRCA1 Associated Protein 1
cmiRNA: Circulating microRNA
cfDNA: Cell-free DNA
CNV: Copy number variation
CSC: Cancer stem cells
CT: Computed tomography
CTCs: Circulating tumor cells
ctDNA: Circulating tumor DNA
D3: Disomy 3
EIF1AX: Eukaryotic Translation Initiation Factor 1A X-Linked
EMT: Epithelial-mesenchymal transition
FACS: Fluorescence-activated cell sorting
FFA: Fundus fluorescein angiography
FFPE: Formalin-fixed paraffin-embedded
FNAB: Fine-needle aspiration biopsy
GEP: Gene expression profiling
GPCR: G protein-coupled receptor
ICB: Immunotherapy like Immune checkpoint blocker
ICGA: Indocyanine green angiography
ITH: Intra-tumoral heterogeneity
LCM: Laser capture microdissection
lncRNAs: Long non-coding rnas
M3: Monosomy 3
MART1: Melanoma antigen recognized by T-cells 1
MCAM: Melanoma cell adhesion molecule
MCSP: Melanoma-associated chondroitin sulfate proteoglycan
MHW-MAA: High-molecular-weight melanoma-associated antigen
MRD: Minimal residual disease
MRI: Magnetic resonance imaging
mUM: Metastatic UM
NGS: Next-generation sequencing
NKT: Natural killer T
OCT: Optical coherence tomography
OS: Overall survival
PDT: Photodynamic Therapy
PET: Positron emission tomography
RT-PCR: Reverse transcriptase-polymerase chain reaction
scRNA-seq: Single-cell RNA sequencing
SF3B1: Splicing Factor 3b Subunit 1
SPECT: Single-photon emission computed tomography
TCGA: The cancer genome atlas
TCIA: The Cancer Immunome Atlas
TCR: T Cell Receptors
TIDE: Tumor Immune Dysfunction and Exclusion
TMB: Tumor mutational burden
TME: Tumor microenvironment
TTT: Transpupillary Thermotherapy
UPS: Ubiquitin–proteasome system
US: Ultrasonography
VISTA: V domain Ig Suppressor T-cell Activation
WES: Whole exome sequencing
WGS: Whole genome sequencing
